# Abnormal Endogenous Repression of GA Signaling in a Seedless Table Grape Cultivar with High Berry Growth Response to GA Application

**DOI:** 10.3389/fpls.2017.00850

**Published:** 2017-05-24

**Authors:** Atiako K. Acheampong, Chuanlin Zheng, Tamar Halaly, Lisa Giacomelli, Yumiko Takebayashi, Yusuke Jikumaru, Yuji Kamiya, Amnon Lichter, Etti Or

**Affiliations:** ^1^Department of Fruit Tree Sciences, Institute of Plant Sciences, Agricultural Research Organization, Volcani CenterBet Dagan, Israel; ^2^Department of Horticulture, Faculty of Agriculture Environment and Food Sciences, The Hebrew University of JerusalemRehovot, Israel; ^3^Research and Innovation Centre-Fondazione Edmund MachSan Michele all’Adige, Italy; ^4^RIKEN Plant Science CenterYokohama, Japan; ^5^Institute of Postharvest and Food Sciences, Department of Postharvest Science of Fresh Produce, Agricultural Research Organization, Volcani CenterBet Dagan, Israel

**Keywords:** DELLA proteins, gene expression, gibberellin, gibberellin signaling, *Vitis vinifera*, VvSLY1

## Abstract

Gibberellin (GA) application is routinely used in the table grape industry to increase berry size and cluster length. Although grapevine cultivars show a wide range of growth responsiveness to GA_3_ application, the reasons for these differences is unclear. To shed light on this issue, two commercial grapevine cultivars with contrasting berry response to GA were selected for comparative analysis, in which we tested if the differences in response: (1) is organ-specific or cultivar-related; (2) will be reflected in qualitative/quantitative differences in transcripts/proteins of central components of GA metabolism and signaling and levels of GA metabolites. Our results showed that in addition to the high response of its berries to GA, internodes and rachis of cv. Black finger (BF) presented a greater growth response compared to that of cv. Spring blush (SB). In agreement, the results exposed significant quantitative differences in GA signaling components in several organs of both cultivars. Exceptionally higher level of all three functional VvDELLA proteins was recorded in young BF organs, accompanied by elevated *VvGID1* expression and lower *VvSLY1b* transcripts. Absence of seed traces, low endogenous GA quantities and lower expression of *VvGA20ox4* and *VvGA3ox3* were also recorded in berries of BF. Our results raise the hypothesis that, in young organs of BF, low expression of *VvSLY1b* may be responsible for the massive accumulation of VvDELLA proteins, which then leads to elevated *VvGID1* levels. This integrated analysis suggests causal relationship between endogenous mechanisms leading to anomalous GA signaling repression in BF, manifested by high quantities of VvDELLA proteins, and greater growth response to GA application.

## Introduction

Unlike seeded grapevine (*Vitis vinifera* L.) cultivars, which have considerably high endogenous GA levels (Lavee, 1960; Kato et al., 1998; Agüero et al., 2000; Perez et al., 2000), berries of the usually small, stenospermocarpic varieties contain low GA quantities since they carry only seed traces, as a result of endosperm abortion following fertilization which leads to cessation of seed development (Iwahori et al., 1967; Cheng et al., 2013). GA application is therefore routinely used to stimulate stenospermocarpic berry development to a commercially acceptable size, and also for rachis stretching and cluster thinning (Weaver, 1958, 1965; Harrell and Williams, 1987). Issues of differential varietal responsiveness to such GA application have been reported in berries and other vegetative tissues/organs, including rachis and shoot (Weaver, 1958; Hagiwara et al., 1980; Mullins et al., 1992; Agüero et al., 2000). For example, while ‘Thompson seedless’ (TS) berries, required 3–4 applications of 30–45 μM of GA for a twofold increase in size (Dreier et al., 1998), application of 290 μM of GA_3_ increased ‘Emperatriz’ berry size by only 20% (Agüero et al., 2000). Similar differences were recorded in response of internodes and rachis (Hagiwara et al., 1980). We previously showed that GA response in grapevine is organ specific (Acheampong et al., 2015), but it is unclear whether varietal differences in GA response is limited to certain tissue/organ types or it is a whole-plant phenomenon.

Varietal differences in response to GA may possibly result from variations in GA signaling components and/or availability of bioactive GA. Studies in model plants have shown that GA activates its response pathway by binding to its receptors, GID1s. This complex then targets DELLAs, the major negative regulators of the GA-response, for degradation by the 26S proteasome through binding with SLY1, GA-specific F-box proteins (Hirano et al., 2008; Sun, 2010, 2011). The grapevine genome encodes three DELLA proteins (VvDELLA1, VvDELLA2, and VvDELLA3), which are redundantly expressed in vegetative and reproductive organs (Acheampong et al., 2015). VvDELLA1 transcripts and proteins were highest in internodes, rachis and tendrils, but were undetected in seeds and berries. Gain-of-function mutation in this gene conferred GA-insensitive phenotype in most organs, but had no effect on berry size (Boss and Thomas, 2002; Chaib et al., 2010). The specific function of VvDELLA2 in grapevine has not been elucidated yet, but its *in vitro* function, its ability to complement orthologous Arabidopsis mutants, and the high transcripts and proteins quantities in most organs of TS suggested a central role for this gene in regulating GA-related physiological processes in grapevine organs. Based on similar functional tests, low abundance in mature organs and higher abundance in developing tissues, it was proposed that VvDELLA3 regulate GA-mediated processes in young organs (Acheampong et al., 2015). Two functional grapevine GID1 homologs, *VvGID1a* and *VvGID1b*, exhibited spatial and temporal expression redundancies, and were down-regulated upon GA application. The two functional VvSLY1 paralogs in grapevine exhibited inverse temporal expression profiles during organ growth and development, and were downregulated by GA (Acheampong et al., 2015).

To investigate the potential involvement of allelic variability or quantitative differences in VvDELLAs, and other GA signaling components, in modulating differences in response to GA between stenospermocarpic grape varieties, we carried out a comprehensive comparative study of their sequence, their nature of interaction and their quantities in cv. Black finger (BF) (Perl et al., 1995), which berries are considered by growers to be highly responsive to GA and cv. Spring blush (SB) (Spiegel-Roy et al., 1994), considered non-responsive to GA. We complemented our study by comparisons of levels of bioactive GAs and of regulatory GA metabolism genes. Our results suggest a central role of VvDELLA accumulation in regulating the varietal differences in response to GA.

## Materials and Methods

### Field Experiment and Sampling

The cultivars selected for this study, BF and SB, are known for consistent differences in response of the berries to GA, in different vineyards of different growers in different regions and growing seasons. Both cultivars are progenies of crosses between varieties in which at least one of the parents is of stenospermocarpic genetic background. BF was obtained from cross between the seeded cv. Barlinka and stenospermocarpic cv. Centennial [cv. GoldB x cv. Q25-6 (F_2_ of cv. Emperor × cv. Sultana muscata)]. SB is a progeny of cv. Superior (stenospermocarpic variety) and cv. Ruby seedless [cv. Emperor × cv. Pirovano 75 (progeny of cv. Muscatel of Alexandria × cv. Thompson seedless)].

All experiments were conducted in the 2010 and replicated in 2011 growing seasons except one experiment (response to GA_1_ and GA_4_), which was carried out in 2013. The experiments were initiated on 10-year-old BF vines in Lachish, Israel (N31°33′33; E34°51′26) and 10-year-old SB vineyard located in Avigdor, Israel (N31°71′19; E34°74′04). These vineyards are only 19 km apart, and present similar topographic and environmental parameters. Vines are grafted on cv. Richter 110 and trained on high cordon suspensions on Y-shaped trellis. The canes were pruned to 15 buds with 6 to 7 canes per vine. Irrigation was by drippers spaced 50 cm apart. Irrigation regime was controlled by the grower according to standard commercial practices, which are evaporation-dependent, and ran between 30 and 80% of evaporation in the spring and summer, respectively. Nitrogen was supplied in the water as 200 kg ha^-1^ of 12% ammonium sulfate from bloom to fruit set, and 1700 L ha^-1^ of 2-2-10 (NPK) solution was supplied in the same manner from fruit set to harvest.

Separate experiments were conducted to examine the effect of applications of bioactive GAs and a GA biosynthesis inhibitor, PAC, on the elongation of internodes and rachis, berry enlargement, and on pistil development. Samples for transcript, protein and GA quantitation were also taken from the same vineyards. To account for possible variations due to geographical location of vineyards, each experiment was compared to the corresponding Triton X-100 control treatments in the same vineyard. To correct for differences due to location of vines in the vineyards, each experiment consisted of three blocks of eight-vine plots, arranged in a randomized complete-block design. Pooled tissues, sampled as outlined below from each block separately, represented a biological replicate. Morphological responses of internodes, rachis and berries to various growth regulators were determined for organs borne on vines in all three blocks.

#### Treatment with Bioactive GAs and PAC

Gibberellin and paclobutrazol treatment regimes of organs are as previously described (Acheampong et al., 2015), and summarized below. For each experiment, three groups of eight uniform 15 cm shoots, and inflorescences with tightly packed flowers [stage 15, E-L 15, on the Modified Eichhorn and Lorenz system (Coombe, 1995)] were selected on vines of similar vigor for internode and rachis, respectively. For pistil experiments, inflorescences with closed flowers (E-L 17) were treated at about 2 weeks before full bloom. Clusters with berries of 2–3 mm diameter (E-L 27) were selected for berry experiments. Organs received a single Triton X-100 (0.025%)-formulated GA_3_ (Pro-Gibb 4%; Abbott Laboratories, Chicago, IL, United States), or 10 mg/L each of GA_1_ and GA_4_ (OlChemIm Ltd, Olomouc, Czechia), or 0.8 mM PAC (CULTAR 25 SC, Syngenta AG, Basel, Switzerland) application. To allow for effective inhibition of GA biosynthesis, PAC was applied 96 h before GA_3_ and Triton X-100 treatments. Accordingly, in PAC-GA treatment, samples received GA_3_ application 96 h after PAC treatment. These PAC-GA treatments were included to verify that the effect of PAC on organ development was mostly GA-biosynthesis related.

##### Morphological response of organs to bioactive GAs and PAC

Internodes, rachises, and berries were treated as described above. Pre-treatment lengths of rachises, and weights of berries were recorded. Increment in length of rachises and the newest internodes arising after treatment, were monitored at 5-day intervals, while berry weights were assessed at 10-day intervals for 40 days. For each treatment regime, rachis and internode measurements were carried out on all 25 treated organs, while berry weight was measure on 150 berries sampled from clusters on vines in all three replicate blocks. For calculation, the initial length of new internodes was assumed to be 0.5 mm.

##### Sampling for GA response and signaling analyses

Organs and tissues (three replicates of eight internodes and rachis, and berries from six clusters, sampled from different vines of each block) were collected 6 and 24 h after GA treatments. The 6 h samplings were before 14:00, whereas the 24 h samplings were before 08:00 of the following day to minimize circadian effects on gene expression.

##### Sampling for temporal and spatial analyses, and GA quantitation

Sampling of young internodes was carried out from the most distal internodes from the base of young shoots at E-L 15, while young rachises were sampled from inflorescence at E-L 15. Internodes and rachises at similar developmental stage were marked and sampled at véraison and were defined as mature internodes and rachis. Young leaves and tendrils were defined as those borne on the 1st and 2nd nodes (from the shoot tip), while mature leaves and tendrils were sampled from the 12th node. Pistils, free from other floral parts, were sampled from inflorescence at E-L 17, while berries (2–3 mm diameter) were sampled at E-L 27, and subsequently at 10 and 30 days after the first sampling; herein referred to as 0, 10, and 30 DAF, respectively. Organs, sampled separately from each block, were pooled and each of the three pools represented the biological replication. Roots were obtained from single node cuttings immersed in water for about 21 days. Samplings were done at 09:00 to minimize circadian effects on gene expression.

### Nucleic Acid Extraction

Genomic DNA (gDNA) was extracted from leaves of BF and SB using modified cetyltrimethyl ammonium bromide (CTAB) methods of Russell et al. (1992). Total RNA was extracted and cDNA synthesized as previously described (Acheampong et al., 2010). Briefly, 3 g of plant material was homogenized in liquid nitrogen and incubated for 10 min at 65°C in CTAB buffer [2% polyvinylpyrrolidone (PVP, 40000), 2% CTAB, 0.1M Tris (pH 8), 25 mM EDTA (pH 8), 2 mM NaCl, 2% β-mercaptoethanol]. The mixture was clarified by centrifuging at 10,000 rpm for 10 min, and the supernatant mixed with Chloroform:Isoamyl alcohol (24:1). The mixture was again centrifuged as above. RNA was precipitated from the aqueous upper layer by mixing with 3.3 M LiCl solution and incubating at 4°C overnight. RNA was pelleted by centrifuging at 10,000 for 20 min, 4°C, and resuspended in SSTE [1 M NaCl, 10 mM Tris (pH 8), 0.5% sodium dodecyl sulfate] solution. Phenol:chloroform:isoamyl alcohol (25:24:1) was added and centrifuged at 13,000 rpm for 5 min. To the upper aqueous layer was added chloroform:isoamyl alcohol (24:1), and centrifuged as above. RNA was precipitated by mixing with two-volumes of ethanol, and incubating at -20°C overnight. RNA was pelleted by centrifuging at 13,000 rpm, 4°C, for 15 min. Pellets were washed with 70% ethanol, and resuspended in 30 μL DNase/RNase-free water. First-strand cDNA was synthesized using Moloney Murine Leukemia Virus Reverse Transcriptase (M-MLV RT) (Promega Corporation, Madison, WI, United States) according to the manufacturer’s instructions.

### Gene Cloning and Plasmid Construction

Full-length ORFs of all genes were PCR-amplified from a mix of cDNA from different organs. Sequences of primers used to clone all genes are listed in Supplementary Table S1. Primers for gene cloning were designed using the web-based Primer3 (ver. 0.4.0) software (Untergasser et al., 2012). PCR fragments to be cloned into Entry vectors were amplified with primers having the recommended recombination overhangs. Cloning of genes for gene-specific polyclonal antibody production, and full-length proteins was as previously described (Acheampong et al., 2015).

For the yeast two-hybrid assay, pGBKT7 and pGADT7 (Clontech, Mountain View, CA, United States) were used as bait and prey expression vectors, respectively. Cultivar-specific alleles of VvGID1s, VvDELLAs, and VvSLY1s were PCR-amplified from cDNA from both cultivars using Phusion High-Fidelity DNA Polymerase (New England Biolabs Inc., Ipswich, MA, United States). cDNA from both VvGID1 and VvSLY1 proteins were expressed in pGBKT7 as fusions with GAL4 DNA binding domain (DNA-BD), while VvDELLA proteins were expressed in pGADT7rec as fusions with the GAL4 activation domain (AD). Cloning into both DNA-BD and AD vectors was carried out using In-fusion HD Cloning Kit (Clontech Laboratories Inc., Mountain View, CA, United States) according to manufacturer’s protocol. In details, the coding sequences of *VvGID1a* and *VvGID1b* were PCR-amplified, with primer sets VvGID1a-1/VvGID1a-2 and VvGID1b-1/VvGID1b-2, respectively. Each *VvGID1* PCR fragment was inserted into pGBKT7 digested with *Eco*RI/*Bam*HI. Likewise, *VvSLY1a* and *VvSLY1b* coding sequences were first PCR-amplified with primer sets VvSLY1a-1/VvSLY1a-2 and VvSLY1b-1/VvSLY1b-2, respectively, and then inserted into pGBKT7 upon *Eco*RI/*Bam*HI digestion. For VvDELLAs, their coding sequences were PCR-amplified, with primer sets VvDELLA1-1/VvDELLA1-2, VvDELLA2-1/VvDELLA2-2, and VvDELLA3-1/VvDELLA3-2, respectively. The PCR products were inserted into pGADT7 after proper restriction digestion using *EcoI*R1/*Bam*HI. Varietal-specific clones, identified after sequencing, were selected and used for Yeast 2-hybrid assays. Primers for yeast two-hybrid cloning were designed using the Clontech Online Tools for In-fusion Cloning^1^, and are listed in Supplementary Table S1.

### Yeast 2-Hybrid Analyses

Yeast 2-hybrid analyses and β-galactosidase liquid assays were carried out as described previously (Boneh et al., 2012), with slight modifications. The cultures were diluted 1:2 with SC-Leu/Trp medium and 10 μL plated onto three selective medium plates: SC-Leu/Trp, SC-Leu/Trp/His with 5 mM 3-amino-1,2,4-triazole (3-AT), and similar plates with 100 μM GA_3_ (for VvDELLA–VvGID1 interactions).

### Quantitative Real-Time PCR (qRT-PCR) Analyses

The transcript levels of *VvGID1*s, *VvDELLA*s, *VvSLY1*s, and *VvGASA*s were measured by quantitative real-time PCR (qRT-PCR) as previously described (Acheampong et al., 2015), while and *VvGA2ox*s, *VvGA3ox*s, and *VvGA20ox*s expressions were by qRT-PCR using EvaGreen DNA-binding dye (Biotium Inc., Hayward, CA, United States) on the 96.96 Dynamic Array Integrated Fluidic Circuits (IFCs) (Fluidigm, San Francisco, CA, United States). Expressions of genes were normalized to transcripts of previously characterized, non-GA regulated *VvGAPDH* (Reid et al., 2006; Giacomelli et al., 2013). Relative transcripts of GA metabolism genes are presented as normalized relative expression (NRE) (Pfaffl, 2001). Whereas *VvGA2ox*s, *VvGA3ox*s, and *VvGA20ox*s expressions were determined for samples collected in the 2010 growing season, all other expression analyses were carried out on tissues collected in both 2010 and 2011 growing seasons. Primers used were designed from nucleotide sequences of accession numbers in Section 2.10, using Primer3 (ver. 0.4.0) software (Untergasser et al., 2012), and are listed in Supplementary Table S1 and our previous publications (Giacomelli et al., 2013; Acheampong et al., 2015).

### Antibody Production

Expression of recombinant proteins in BL21-CodonPlus (DE3) RIPL strains (Strategene, Santa Clara, CA, United States), and production of polyclonal antibodies was previously detailed (Acheampong et al., 2015). Full-length VvDELLA proteins, used as sizing standards to locate endogenous VvDELLA proteins, were also expressed and purified as previously described (Acheampong et al., 2015), and quantified using BSA standards.

### Protein Extraction and Immunoblot Analyses of VvDELLA Proteins

Total protein was extracted from samples collected in both 2010 and 2011 growing seasons by the previously described protocol of Wang et al. (2006) with slight modifications. Samples (0.5 g) were first homogenized in liquid nitrogen in the presence of polyvinylpolypyrrolidone (PVPP). The protein pellets obtained were dissolved in SDS-PAGE sample buffer containing 0.15 M Tris (pH 6.8), 1.2% SDS, 30% glycerol, 2.14 M β-mercaptoethanol (Sigma-Aldrich, St Louis, MO, United States). Extracted proteins were quantified by band intensities confirmed by fractionating on 10% SDS-PAGE gel, and staining with Coomassie protein staining buffer (0.1% Coomassie Brilliant Blue R-250, 50% methanol and 10% glacial acetic acid). Equal amounts of proteins were separated by 10% SDS-PAGE and transferred to PROTEAN nitrocellulose transfer membrane (Whatman GmbH, Dassel, Germany) using the Mini-Protean Transfer system (Bio-Rad Laboratories, Hercules, CA, United States). Immunoblot assays are as previously described (Acheampong et al., 2015). Band intensities were analyzed using ImageJ 1.48v software (Schneider et al., 2012).

### Quantitation of Endogenous Gibberellins

Extraction and quantitation of endogenous gibberellins from tissues collected in 2010 growing season were carried as previously described (Acheampong et al., 2015). Quantities of ^2^H_2_-labeled GA species used as the Internal Standard are provided in Supplementary Table S2.

### Statistical Analyses

Unless otherwise stated, all experiments were conducted in a completely randomized block designs. Data are presented in tables and bar graphs as the mean ± standard error (SE) or standard deviation (SD). Statistical significance for gene expression data between cultivars was determined by Student’s *t*-test, whereas differences among organs of a cultivar were analyzed by one-way Analysis of Variance (ANOVA) followed by Tukey HSD multiple comparison tests (JMP 13.1.0 software, SAS Institute, Cary, NC, United States), and significant values set at α = 0.05 (see **Supplementary File S2**). For GA metabolism genes, the ANOVA and Student’s *t*-test analyses were performed on log_2_(NRE). Data from field experiments was also statistically analyzed by ANOVA (α = 0.05).

### Accession Numbers

GenBank Accession numbers of sequences referred to in this study were obtained from the Genoscope^2^) predictions of the 12x genome of *V. vinifera* libraries, and are as follows: VvGID1a (GSVIVT01022014001), VvGID1b (GSVIVT01011037001), VvDELLA1 (GSVIVT01011710001), VvDELLA2 (GSVIVT01030735001), VvDELLA3 (GSVIVT01015465001), VvSLY1a (GSVIVT01000213001), VvSLY1b (GSVIVT01009408001), VvGASA1 (GSVIVT01009902001), VvGASA2 (GSVIVT01011412001), VvGASA3 (GSVIVT01033563001), VvGASA4 (GSVIVT01008003001), VvGASA5 (GSVIVT01009384001), VvGASA6 (GSVIVT01034477001).

The cultivar-specific alleles of GA signaling genes cloned and analyzed in this study, have been deposited on NCBI with the following accession numbers: VvDELLA1_BF (KY765590), VvDELLA1_SB (KY765591), VvDELLA2_BF (KY765592), VvDELLA2_SB (KY765593), VvDELLA3_BF_1 (KY765594), VvDELLA3_BF_2 (KY765595), VvDELLA3_SB_1 (KY765596), VvDELLA3_SB_2 (KY765597), VvGID1a_BF (KY765598), VvGID1a_SB (KY765599), VvGID1b_BF_1 (KY765600), VvGID1b_BF_2 (KY765601), VvGID1b_SB_1 (KY765602), VvGID1b_SB_2 (KY765603), VvSLY1a_BF (KY765604), VvSLY1a_SB (KY765605), VvSLY1b_BF (KY765606), VvSLY1b_SB_1 (KY765607), VvSLY1b_SB_2 (KY765608).

## Results

### Response of Organs of BF and SB to Exogenous GA_3_ and PAC

The cultivars selected for this study, BF and SB, are known for reproducible differences in response of the berries to GA, in different vineyards of different growers, in different regions and years. To investigate the scope of varietal differences in grapevines, we carried out a comparison of the responses of vegetative and reproductive organs of BF and SB to application of predetermined, informative concentration of GA_3._ For these analyses, which required many mature vines, we selected the closest BF and SB vineyards available, with very similar topographic and environmental parameters, and carried out the analysis over two consecutive growing seasons.

**FIGURE 1:**
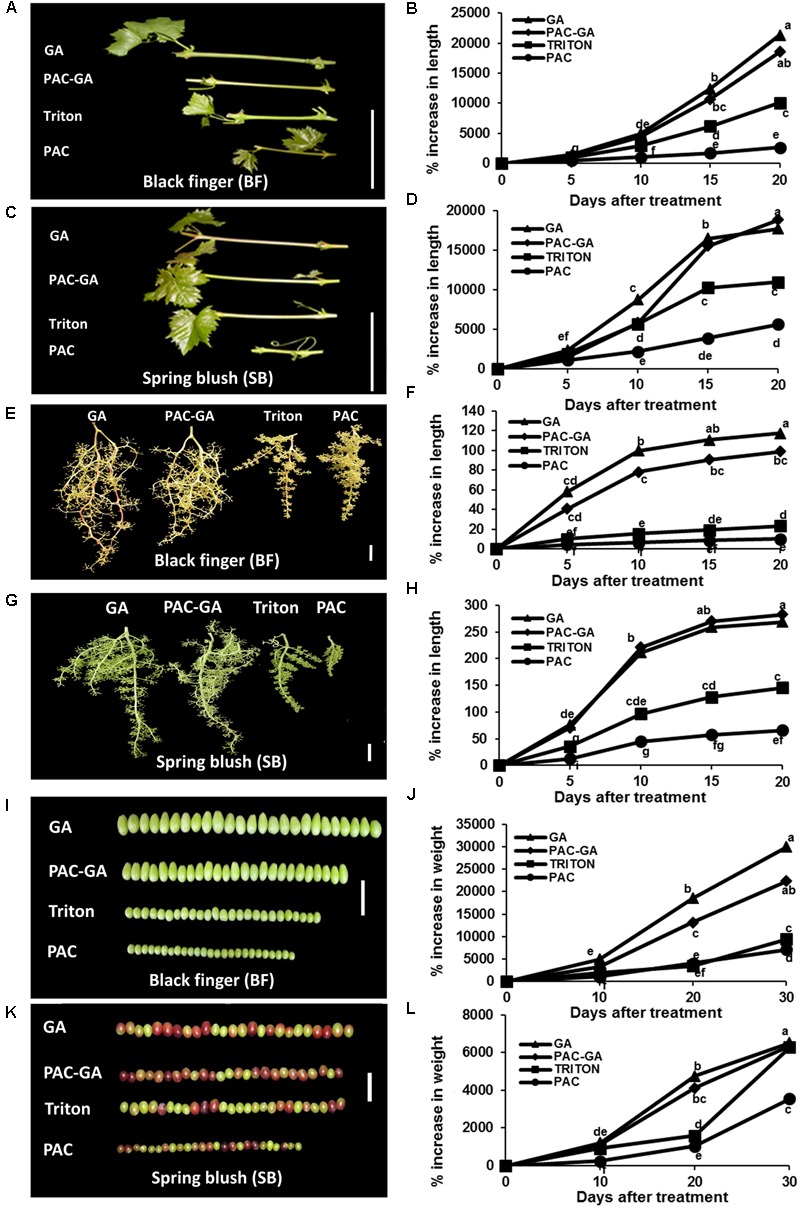
**Effect of GA_3_ and PAC on size of organs of *V. vinifera* cv. Black finger (BF) and cv. Spring blush (SB).** Altered response of organs of BF **(A,B,E,F,I,J)** and SB **(C,D,G,H,K,L)** to GA_3_ and GA biosynthesis inhibitor, PAC treatments during the 2010 growing season (similar results were obtained in experiments carried out in 2011 growing season). GA_3_ and PAC (0.8 mM) included Triton X-100 (0.025%). Internodes and rachises were treated with 121 μM GA_3_, while berries were treated with 90 μM GA_3_. Tissues/organs were dipped or sprayed until run-off. Increase in size was monitored at specific time intervals. Young shoots and inflorescences with tightly packed flowers (stage 15, E-L 15, on the Modified Eichhorn and Lorenz system) were selected for internodes and rachis experiments, respectively. Clusters with berries of 2–3 mm diameter (E-L 27) were selected for berry experiments. **(A,C)** Gross morphology of representative internodes of BF and SB after 20 days of treatment. **(E,G)** Gross morphology of representative rachises of BF and SB after 20 days of treatment. **(I,K)** Gross morphology of representative berries of BF and SB after 30 days of treatment. **(B,D)** Average per cent increase in length of new internodes arising after treatment. Increase in length of internode is expressed as per cent increase of initial length, which was assumed to be 0.5 mm. **(F,H)** Average per cent increment (as a factor of pre-treatment length) in length of rachises of BF and SB. **(J,L)** Average per cent increase in berry weight relative to mean weight at time of treatment (0 day). Data points with different letters indicate significantly different values according to Tukey HSD LSMean test at α = 0.05 and 25 measurements, except for berries with 150 measurements. Bar = 5 cm.

Since GA signaling and metabolism occur predominantly in young, growing tissues (Silverstone et al., 1997; Chandler et al., 2002; Kaneko et al., 2003), we restricted our comparative analyses to only young organs of both cultivars. Compared to the control, GA_3_ treatments resulted in 2.1- and 1.6-fold increase in internode elongation of BF and SB, respectively, while PAC treatments caused a 3.7- and 2-fold reduction in internode length of both cultivars (**Figures 1A–D**). Whereas GA_3_ treatment produced 5- and 1.8-fold increases in rachis lengths of BF and SB, respectively, PAC treatment resulted in approximately 2-fold reduction in both cultivars (**Figures 1E–H**). In contrast, GA_3_ application yielded a 3-fold increase in berry weight of BF, but did not significantly alter berry size of SB (**Figures 1I–L**). PAC treatment led to a 2-fold decrease in berry weight of SB, and a 1.3-fold change in berry weight of BF. To verify that the effect of PAC (which may also affect ABA biosynthesis) was mostly GA-biosynthesis related, PAC-GA treatments were included, in which GA_3_ was applied 96 h after PAC treatment. The PAC-GA data show that, for all organs of both cultivars, the effect of PAC was either partially or fully rescued by the GA treatment. It is interesting to note that at 20 days after treatment, the growth rate of triton-treated control rachis of SB was more than 5-fold greater than corresponding rachis of BF (**Figures 1E–H**). Growth rate of triton-treated internodes and berries are relatively similar for both cultivars after 20 and 30 days, respectively (**Figures 1A–D,I–L**).

### Response of *VvGASA* Genes to Exogenous GA_3_ and PAC

Some Arabidopsis *GASA* genes exhibit markedly different responses to GA_3_ (Aubert et al., 1998), and function downstream of DELLA and PIFs to regulate GA signaling and response (Zhang and Wang, 2008). To evaluate whether the varietal differences in GA response described above result from factors downstream or upstream of *VvGASA* genes, we analyzed *VvGASA* transcripts in GA_3_- and PAC-treated rachis and berries of BF and SB (Supplementary Figure S1). In rachis of BF, PAC treatment increased *VvGASA1* transcripts by 2.5-fold but reduced *VvGASA2* and *VvGASA3* expressions by 1.8- and 2-fold, respectively, and had no effect on *VvGASA4, VvGASA5*, and *VvGASA6* expressions. Similarly, rachis of SB exhibited upregulation of *VvGASA1* transcripts by 3.7-fold but no change in expression of the other *VvGASA* paralogs. Application of GA_3_ to rachis of BF increased *VvGASA3* and *VvGASA4* expressions by 3.2- and 2.4-fold, respectively, but had no effect on other *VvGASA* genes. GA_3_ treatment increased *VvGASA1, VvGASA3*, and *VvGASA4* transcripts in SB rachis by 3.7-, 3-, and 4-fold, respectively, reduced *VvGASA5* expression by 2.6-fold and had no effect on expression of *VvGASA6.*

Paclobutrazol treatment of BF berries had no effect on expression of *VvGASA* genes, except *VvGASA4* and *VvGASA5* whose expressions were 1.7-fold down- and upregulated, respectively. Conversely, PAC application to berries of SB increased *VvGASA1, VvGASA5*, and *VvGASA6* transcript by 7-, 1.7-, and 2.7-fold, respectively, and reduced *VvGASA3* transcript by 1.5-fold. When berries of BF were treated with GA_3_, expression of *VvGASA2, VvGASA3*, and *VvGASA4* was increased by 1.7-, 4-, and 6-fold, respectively, while *VvGASA1, VvGASA5*, and *VvGASA6* expressions were decreased by approximately 2-fold. Similar treatment to SB berries resulted in 2-fold increase in *VvGASA2* and *VvGASA6* transcripts, and a 2.8-fold upregulation of *VvGASA4* expression.

The significant varietal differences of *VvGASA* expressions in response to GA may indicate that varietal differences in organ response is mediated by factors upstream of the *VvGASA* genes. Thus, similar to other plants in which GA signaling regulates GA-related plant growth (Achard and Genschik, 2009), it is hypothesized that variations in response to GA_3_ may, at least partly, be determined by qualitative and/or quantitative variations of the signaling components.

### Allelic Variations Did Not Influence Interactions between GA Signaling Components

To check whether the differences in GA response between BF and SB are the result of allelic variation that may affect the quality of interaction and hence GA-mediated VvDELLA degradation, all alleles of the previously characterized grapevine VvDELLAs, VvGID1s, and VvSLY1s (Acheampong et al., 2015) in both BF and SB were sequenced. Functional interactions between the GA signaling components were also analyzed by Y2H assays. Nucleotide sequence analyzes showed cultivar-specific point mutations in *VvGID1a, VvGID1b, VvSLY1a*, and *VvSLY1b* genes (Supplementary Figures S2A–D). Yet, these mutations did not result in changes in coded amino acids as these amino acid sequences were similar to previously sequenced genes of TS (Acheampong et al., 2015). There were no cultivar-specific differences in the nucleotide or amino acid sequences of VvDELLA1. However, there were substitutions in sequences of *VvDELLA2* of both cultivars at positions 22 (A-G substitution), 35 (C-G substitution), 1161 (A-G substitution), and 1426 (C-T substitution) (Supplementary Figure S2E). The first two resulted in S^8^G (Ser at position 8 of BF replaced by Gly in SB), and A^12^G (Ala at position 12 replaced by Gly) substitutions (Supplementary Figure S2F). Deletion of nucleotide sequence GGC (number 46-48) in *VvDELLA2* of BF, compared to SB, resulted in the in-frame deletion of Gly at position 16. Two alleles of *VvDELLA3* differed between the cultivars in nucleotide substitutions G-C at positions 38 and 394, A-T at position 390, A-C at position 1092 and 1538, and T-C substitution at 1320 (Supplementary Figure S2G). While sequence variations between alleles of BF did not result in changes in amino acids, there was S^13^T (Ser–Thr) amino acid substitutions at position 13 of SB. Comparing BF ORF to ORFs of both alleles of SB, there were Ser–Thr and Glu–Gln substitutions at positions 13 (S^13^T) and 132 (E^132^Q), respectively (Supplementary Figure S2H).

**FIGURE 2:**
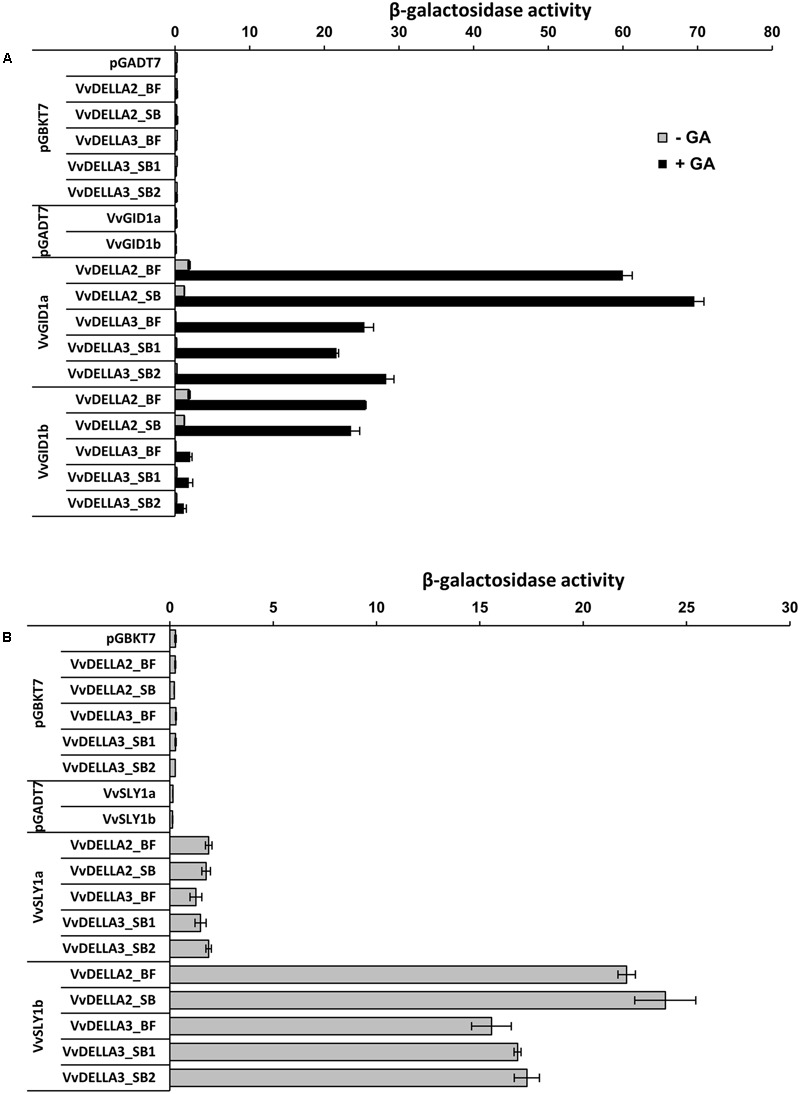
**Different alleles of VvDELLAs isolated from cv. Black finger (BF) and cv. Spring blush (SB) interact with VvGID1s and VvSLY1s in Y2H assays. (A)** Interaction between VvDELLAs and VvGID1s proceed in a GA-dependent manner. The addition of 100 μM GA_3_ to the medium enhanced GID1–DELLA interactions. **(B)** Interaction between VvDELLAs and VvSLY1s. VvDELLA3_SB1 and VvDELLA3_SB2 represent the two alleles of VvDELLA3 isolated from SB. The bars represent the mean ± SE of at three replicates.

These varietal changes in amino acid sequences of VvDELLA2 and VvDELLA3 did not result in significant differences in strength of interaction with VvGID1s (**Figure 2A**) or VvSLY1s (**Figure 2B**) in Y2H assays. Similar to clones of TS (Acheampong et al., 2015), both VvGID1 homologs interacted with each of the VvDELLA2 clones from both cultivars in a GA-dependent manner, while VvGID1b did not interact with any of the VvDELLA3 alleles, even in the presence of GA_3_. Compared to VvSLY1a, VvSLY1b interacted stronger with all VvDELLA homologs and alleles.

Results of qRT-PCR analyses show that there was no obvious effect of GA_3_ or PAC treatments on the expressions of all three *VvDELLA* genes in both cultivars after 6 and 24 h (Supplementary Figure S3). *VvDELLA1*, which did not have any sequence variation between the cultivars, was included in this analysis as a control. It should be noted that the *VvDELLA3* expression results represent total *VvDELLA3* expression of both alleles.

### Organs of BF, the Cultivar with Higher Response to GA_3_, Accumulated Remarkably Higher Levels of VvDELLA Proteins

Since both loss-of-function and gain-of-function DELLA mutants display impaired GA signaling and are defective in GA response (Dill et al., 2001; Ikeda et al., 2001; King et al., 2001; Boss and Thomas, 2002), we assumed that varietal differences in VvDELLA quantities may result in differences in response to GA: a variety with higher VvDELLA quantities will exhibit greater growth repression and subsequently higher response to GA application, compared to variety with lower quantities. Accordingly, transcript and protein levels of the previously characterized *VvDELLA*s paralogs were determined in both cultivars.

**FIGURE 3:**
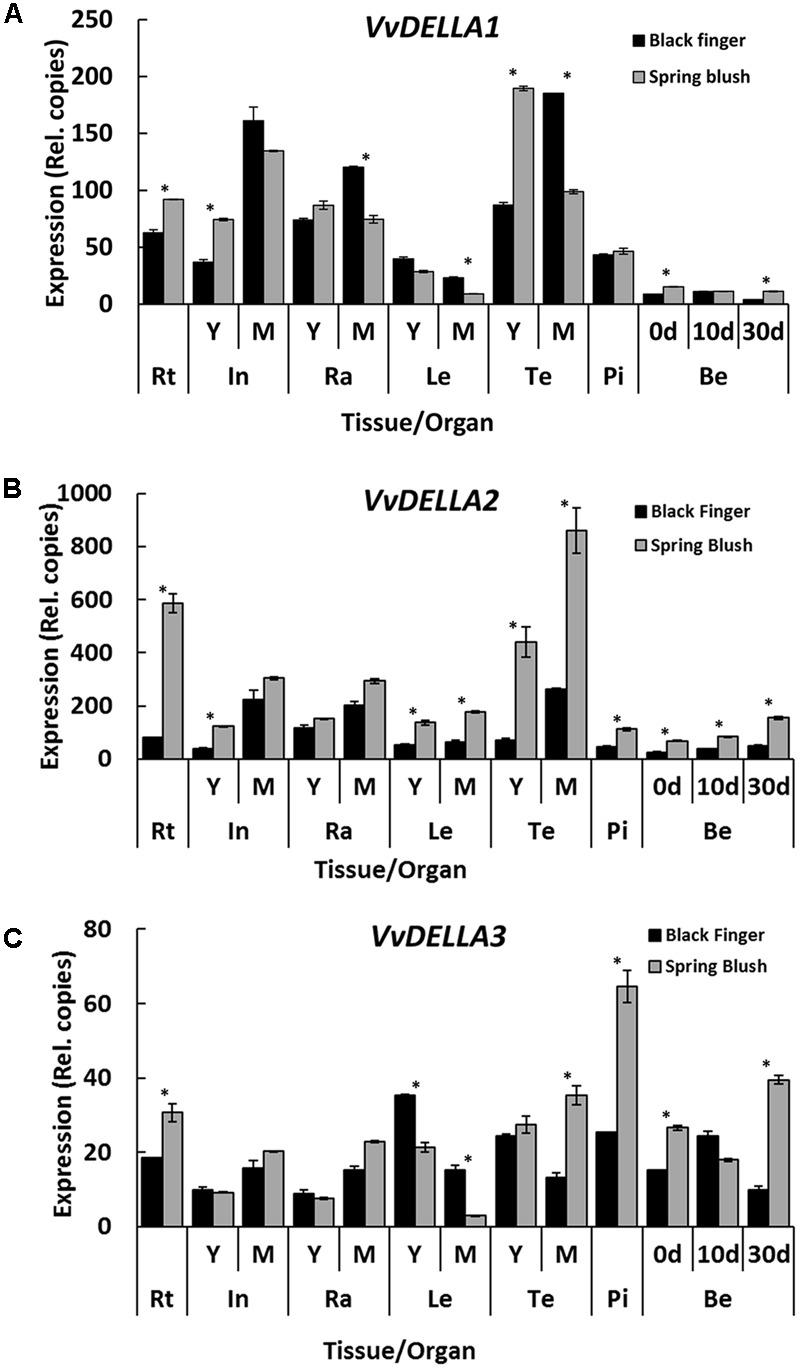
**Spatio-temporal expression profile of *VvDELLA* paralogs in *V. vinifera* cv. Black finger (BF) and cv. Spring blush (SB).** Spatial and temporal expression profiles of *VvDELLA1* **(A)**, *VvDELLA2* **(B)**, and *VvDELLA3* **(C)** in BF and SB organs sampled during the 2010 growing season. Total RNA was extracted from pooled samples, and the absolute mRNA levels of each gene were determined by real-time quantitative RT-PCR (qRT-PCR) and normalized against *VvGAPDH*. To ensure accurate quantitation of transcript levels, primers of similar efficiencies were used, and calibration curves determined from known copy numbers of single plasmid containing all qRT-PCR amplicons. The bars represent the mean ± SE. of three biological repeats with two technical repeats each. Asterisks (^∗^) indicates relative expression levels that are significantly different (Student’s *t*-test; *P* < / > 0.05) between both cultivars. Statistical significance of relative expression values among organs of individual cultivars were calculated using Tukey HSD LSMean test at α = 0.05, and presented in **Supplementary File S2**. In, internodes; Ra, rachis; Le, leaves; Te, tendrils; Pi, pistils; Be, berries; 0 d, berries sampled at 2–3 mm diameter (E-L 27); 10 d, berries sampled 10 days after E-L 27; 30 d, Berries sampled 30 days after E-L 27; Y, young; M, mature. The experiment was repeated during 2011 growing season.

Similar to other grapevine cultivars (Boss and Thomas, 2002; Acheampong et al., 2015), the *VvDELLA*s were expressed in all organs of BF and SB (**Figure 3**). As previously described for TS (Acheampong et al., 2015), *VvDELLA1* and *VvDELLA2* were the most predominant homologs in both BF and SB. Generally, *VvDELLA2* and *VvDELLA3* transcripts were higher in SB compared to BF organs of similar developmental stage. The only exceptions were in internodes, rachis and berries at 10 DAF where *VvDELLA3* expressions were similar in both organs, and in young and mature leaves where *VvDELLA3* was 1.5- and 4-fold higher in BF.

**FIGURE 4:**
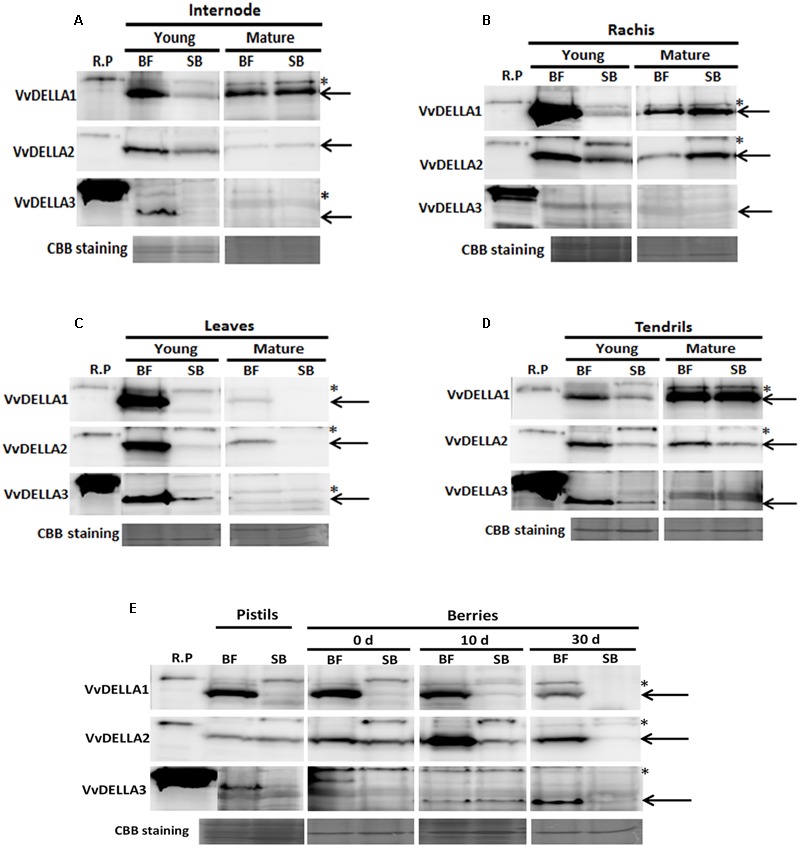
**Spatio-temporal profile of VvDELLA proteins in *V. vinifera* cv. Black finger (BF) and cv. Spring blush (SB).** Blots of total protein extracted from internodes **(A)**, rachises **(B)**, leaves **(C)**, tendrils **(D)**, pistils and berries **(E)** at different developmental stages. Sampling of the tissues was carried out in 2010. Blots were incubated with affinity-purified, gene-specific, anti-VvDELLA polyclonal antibodies. Recombinant full-length proteins (R.P.) (3.75 ng each of VvDELLA1 and VvDELLA2 and 37.5 ng of VvDELLA3) were used as sizing controls. Coomassie Brilliant Blue-stained (CBB) proteins were used as loading control. In all lanes except R.P., solid black arrows show band of interest, and asterisked-bands (^∗^) indicate non-specific proteins detected by the anti-VvDELLA antibodies. Differences in sizes of R.P. and endogenous VvDELLA proteins result from V5 and 6xHis tags on the R.P. 0 d, berries sampled at 2–3 mm diameter (E-L 27); 10 d, berries sampled 10 days after E-L 27; 30 d, berries sampled 30 days after E-L 27. Similar results were obtained when samples collected in 2011 growing season were analyzed.

The fact that in grapevines and other model plants DELLA is mostly regulated by its protein turnover and not transcript quantities (Dill et al., 2004; Arana et al., 2011; Acheampong et al., 2015), prompted us to determine quantities of VvDELLA proteins in both cultivars. Results of immunoblot analyzes, using the gene specific anti-VvDELLA polyclonal antibodies show considerably higher levels of the three VvDELLA proteins in all young organs of BF, compared to SB organs at similar developmental stage (**Figure 4**).

Levels of VvDELLA1, VvDELLA2, and VvDELLA3 proteins were 20-, 4-, and 38-fold higher in young internodes of BF, compared to SB (**Figure 4A**), and decreased during BF internode development. In general, VvDELLA levels in mature internodes were similar in both varieties. VvDELLA1 and VvDELLA2 were 38- and 6-fold higher in young rachis of BF compared to SB, whereas VvDELLA3 was not detected in both young and mature rachis of both varieties (**Figure 4B**). VvDELLA1 and VvDELLA2 protein level decreased during BF rachis maturation, but in SB the protein quantities of these genes increased, and were 2- and 22-fold higher in mature rachis of SB compared to BF.

While high levels of VvDELLA1 and VvDELLA2 proteins were detected in young leaves of BF, these proteins were not detected in young SB leaves (**Figure 4C**). VvDELLA3 was 16-fold higher in young leaves of BF compared to SB. Generally, VvDELLA levels decreased as leaves of both cultivars mature. All three VvDELLA proteins were not detected in mature leaves of SB. Similar to most organs, VvDELLA1, VvDELLA2, and VvDELLA3 were 3-, 5-, and 11-fold higher in young tendrils of BF than SB (**Figure 4D**). Whereas VvDELLA1 accumulated during tendril development of both cultivars, the quantities of VvDELLA2 were unchanged, while VvDELLA3 reduced. VvDELLA1 was similar in both mature tendrils of BF and SB, while VvDELLA2 was 4-fold higher in BF than SB. VvDELLA3 was not detected in mature rachis of both cultivars.

VvDELLA1 was present in substantially high level in BF pistils but was barely detected in pistils of SB (**Figure 4E**). VvDELLA2 and VvDELLA3 quantities were similar in pistils of both cultivars. While VvDELLA1 was not detected in berries of SB, significantly high levels of the protein was present throughout berry development of BF. VvDELLA2 was present in both BF and SB berries but was significantly higher in BF berries at all analyzed time points (8-, 73-, and 203-fold, respectively, higher in BF in berries at 0, 10, and 30 DAF). Interestingly, whereas VvDELLA2 protein accumulation was low and gradually decreased during SB berry development, the quantities of this protein in berries of BF peaked at 10 DAF. VvDELLA3 was undetected in both cultivars at 0 DAF, similar at 10 DAF, but was 30-fold higher in BF at 30 DAF. Similar to VvDELLA2 in BF, the levels of VvDELLA3 in berries of SB was highest at 10 DAF, while the levels of the protein in BF progressively increased during development of BF berries.

**FIGURE 5:**
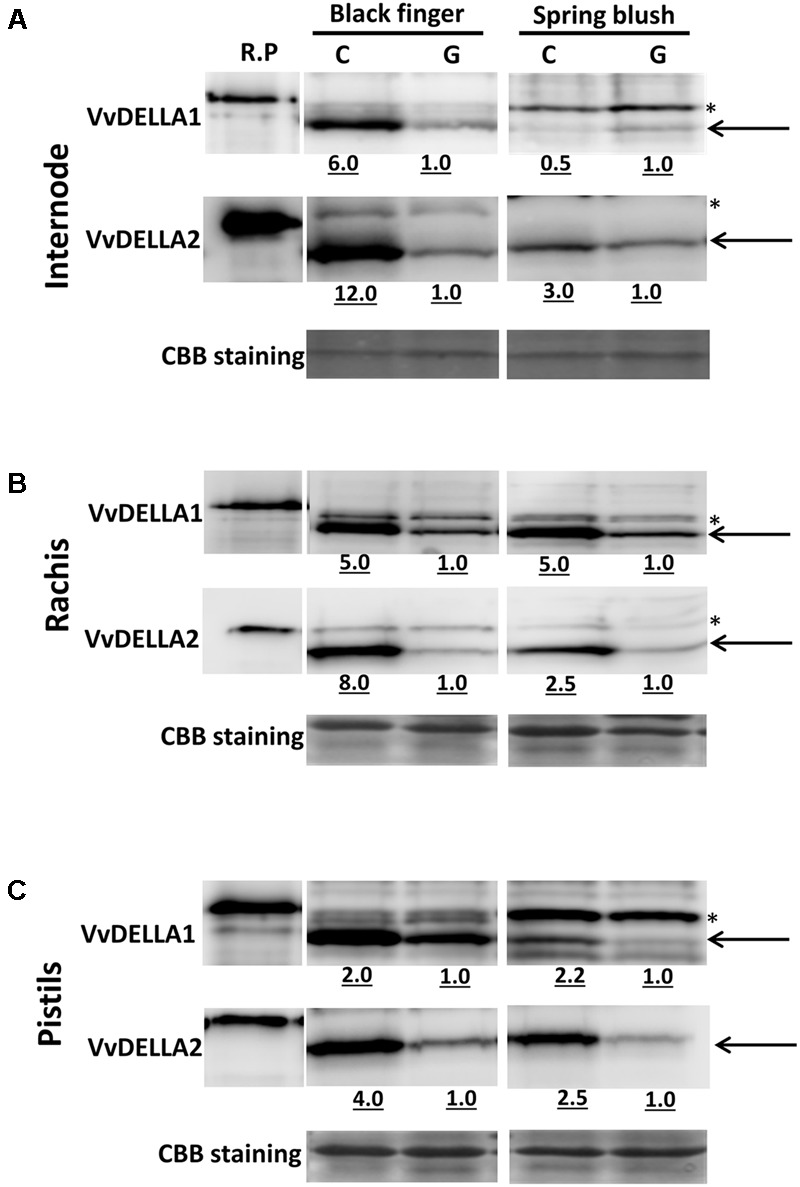
**Effect of GA_3_ application on VvDELLA accumulation in *V. vinifera* cv. Black finger (BF) and cv. Spring blush (SB).** GA_3_-induced degradation of VvDELLA1 and VvDELLA2 proteins in internodes **(A)**, rachis **(B)**, and pistils **(C)** of BF and SB collected during the 2010 growing season. Immunoblot analyzes of VvDELLA proteins in organs were carried out using protein-specific, affinity-purified, anti-VvDELLA polyclonal antibodies. Total proteins were extracted from organs treated for 6 h with GA_3_ (G, 121 μM for rachis, and 90 μM for pistils). Control (C) samples were treated with Triton X-100 (0.025%). Physiological stage at which organs were treated is detailed in “Materials and Methods.” Recombinant full-length proteins (R.P.) (3.75 ng each of VvDELLA1 and VvDELLA2) were used as size controls. In all lanes except R.P., solid black arrows show band of interest, and Asterisked-bands indicate non-specific proteins detected by the anti-VvDELLA antibodies. Differences in sizes of R.P. and endogenous VvDELLA proteins result from tags on the R.P. Underlined numbers indicate intensity of bands relative to GA_3_-treated samples as determined by ImageJ. Consistent results were obtained when the experiment was repeated during the 2011 growing season.

One potential explanation for the higher levels of DELLA proteins in BF was that GA signaling and GA-dependent proteolysis of VvDELLA is impaired in young organs of BF. As both exogenous and endogenous bioactive GAs regulate DELLA proteins accumulation and *GID1* transcripts by negative feedback mechanism in grapevine and other species (Ueguchi-Tanaka et al., 2005; Griffiths et al., 2006; Voegele et al., 2011; Lange et al., 2012; Acheampong et al., 2015), we evaluated the possibility of altered GA signaling by analyzing the levels of *VvGID1*s transcripts and VvDELLA proteins in BF and SB in response to GA. In accordance with the negative feedback regulation, GA application downregulated *VvGID1* transcripts, while PAC upregulated expression of the genes in organs of both cultivars (Supplementary Figures S4A–D). Similar results were obtained in the 2011 growing season (data not shown). Immunoblot analyses of young organs also show that both VvDELLA1 and VvDELLA2 proteins were significantly reduced in response to GA (**Figure 5**). Whereas VvDELLA1 was very low in internodes of SB, it was very high in untreated internodes of BF. GA treatment caused a 6-fold reduction in levels of this protein in internodes of BF. The same treatment resulted in 12- and 3-fold reduction in VvDELLA2 quantities in internodes of BF and SB, respectively. Similar GA treatments also led to significant reduction in protein levels of VvDELLA1 and VvDELLA2 in rachis and pistils of both cultivars. Due to a limiting amount of sampled tissues, similar *in planta* assay could not be conducted to ascertain the GA-dependent VvDELLA3 degradation. While these results suggest that GA signaling and GA-dependent proteolysis of VvDELLA are functional in both varieties, they do not exclude potential differences in efficiency of such proteolysis.

To investigate other sources for the observed varietal differences in VvDELLA accumulation, we analyzed factors such as mRNA quantities of *GID1*s and *SLY1*s, and levels of endogenous GAs; all of which mediate changes in DELLA proteolysis/accumulation in different species (Dill et al., 2001; King et al., 2001; McGinnis et al., 2003; Sasaki et al., 2003; Griffiths et al., 2006; Willige et al., 2007; Li et al., 2011).

### BF, the Cultivar with Higher Response to GA_3_, Had Lower Level of *VvSLY1b* Transcript

**FIGURE 6:**
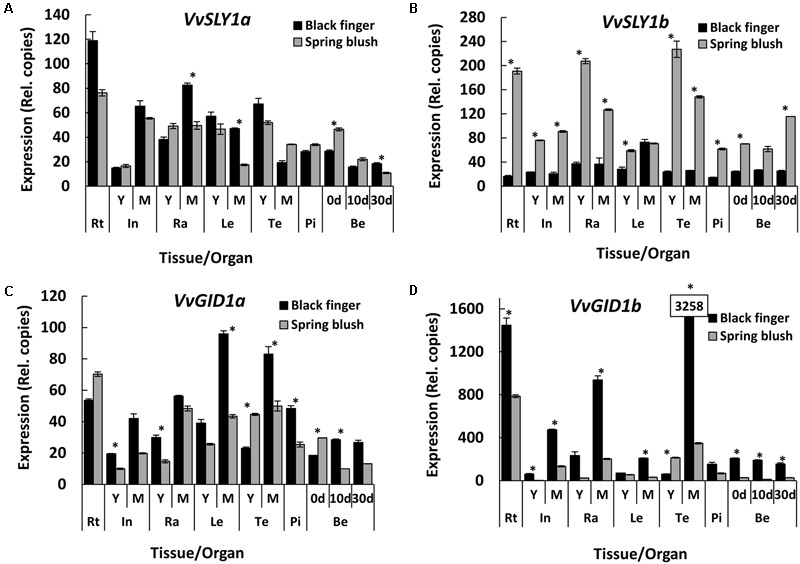
**Spatio-temporal expression profiles of *VvGID1* and *VvSLY1* paralogs in *V. vinifera* cv. Black finger (BF) and cv. Spring blush (SB).** Spatial and temporal expression profiles of *VvSLY1a* **(A)**, *VvSLY1b* **(B)**, *VvGID1a* **(C)**, and *VvGID1b* **(D)** in BF and SB organs collected during the 2010 growing season. Total RNA was extracted from pooled samples, and the absolute mRNA levels of each gene were determined by real-time quantitative RT-PCR (qRT-PCR) and normalized against *VvGAPDH*. To ensure accurate quantitation of transcript levels, primers of similar efficiencies were used, and calibration curves determined from known copy numbers of single plasmid containing all qRT-PCR amplicons. The bars represent the mean ± SE. of three biological repeats with two technical repeats each. Asterisks (^∗^) indicates relative expression levels that are significantly different (Student’s *t*-test; *P* < / > 0.05) between both cultivars. Statistical significance of relative expression values among organs of individual cultivars were calculated using Tukey HSD LSMean test at α = 0.05, and presented in **Supplementary File S2**. In, internodes; Ra, rachis; Le, leaves; Te, tendrils; Pi, pistils; Be, berries; 0 d, berries sampled at 2–3 mm diameter (E-L 27); 10 d, berries sampled 10 days after E-L 27; 30 d, berries sampled 30 days after E-L 27; Y, young; M, mature. Similar results were obtained from samples collected during the 2011 growing season.

Young organs/tissues of SB generally presented higher *VvSLY1* transcripts than corresponding organs of BF (**Figures 6A,B**). Compared to BF, *VvSLY1b* transcript was 3-fold higher in young internode, pistils and young berries of SB, and 6-, 2-, and 12-fold higher in young rachis, leaves and tendrils of SB. *VvSLY1b* in mature internodes, rachis and tendrils, was 4-, 3-, and 6-fold, respectively, higher in SB, compared to BF. *VvSLY1a* expression was, however, only marginally higher in SB pistils (1.2-fold), young rachis (1.2-fold) and berries at 0 and 10 days (1.6- and 1.4-fold, respectively), and slightly lower in young leaves and tendrils of SB (0.2- and 0.3-fold, respectively). However, with the exception of mature tendrils, *VvSLY1a* was lower in mature organs of SB than BF, with leaves recording the highest differences of 3-fold. It is worth-noting that both cultivars displayed the inverse temporal expression profiles of *VvSLY1* homologs, similar to the previously described profile of TS (Acheampong et al., 2015).

Since SLY1 is a central regulator of DELLA proteins degradation (McGinnis et al., 2003; Sasaki et al., 2003; Dill et al., 2004; Sun, 2010), the results raise the possibility that higher accumulation of all three VvDELLAs, detected in BF, may be the consequence of lower levels of their common regulator, *VvSLY1b*, and hence lower efficiency of DELLA degradation in the untreated organs. Interestingly, Y2H assays show stronger interactions between VvSLY1b and VvDELLA genes cloned from BF and SB, compared to VvSLY1a (Acheampong et al., 2015) (**Figure 2B**).

### BF, the Cultivar with Higher Response to GA_3_ Had Higher Level of *VvGID1* Transcript

As DELLA proteins were increased in *gid1* mutants of rice and Arabidopsis (Ueguchi-Tanaka et al., 2005; Griffiths et al., 2006; Willige et al., 2007), we explored the possibility that the varietal differences in VvDELLA accumulation may result from differences in expression of *VvGID1* in the cultivars. We found higher levels of *VvGID1* expression in organs of BF, compared to SB (**Figures 6C,D**). The only exceptions were in young tendrils, where *VvGID1a* (**Figure 6C**) and *VvGID1b* (**Figure 6D**) were 2- and 3-fold higher in SB, respectively. For most organs, there was higher expression of *VvGID1b*, and varietal difference in expression was higher for *VvGID1b* than *VvGID1a*. *VvGID1a* and *VvGID1b* mRNA quantities in young internodes of BF were 2- and 20-fold higher than in SB. Similarly, *VvGID1*a and *VvGID1b* expression in rachis of BF was 2- and 10-fold, respectively, greater than in SB. Transcript levels of *VvGID1a* in young berries (10–30 DAF) of BF were at least 2-fold higher than in SB berries at similar stage, and *VvGID1b* was 6-fold higher in BF at all developmental stages of berries. In light of the above, a possibility was raised that a greater number of GA receptors in BF may contribute to increased GA response in this cultivar, due to increased number of GA-VvGID1-VvDELLA complexes upon GA application, resulting in higher efficiency of VvDELLA degradation.

### SB, the Cultivar with Lower Response to Exogenous GA_3_, Had Higher Levels of GA_4_ in Developing Berries

**FIGURE 7:**
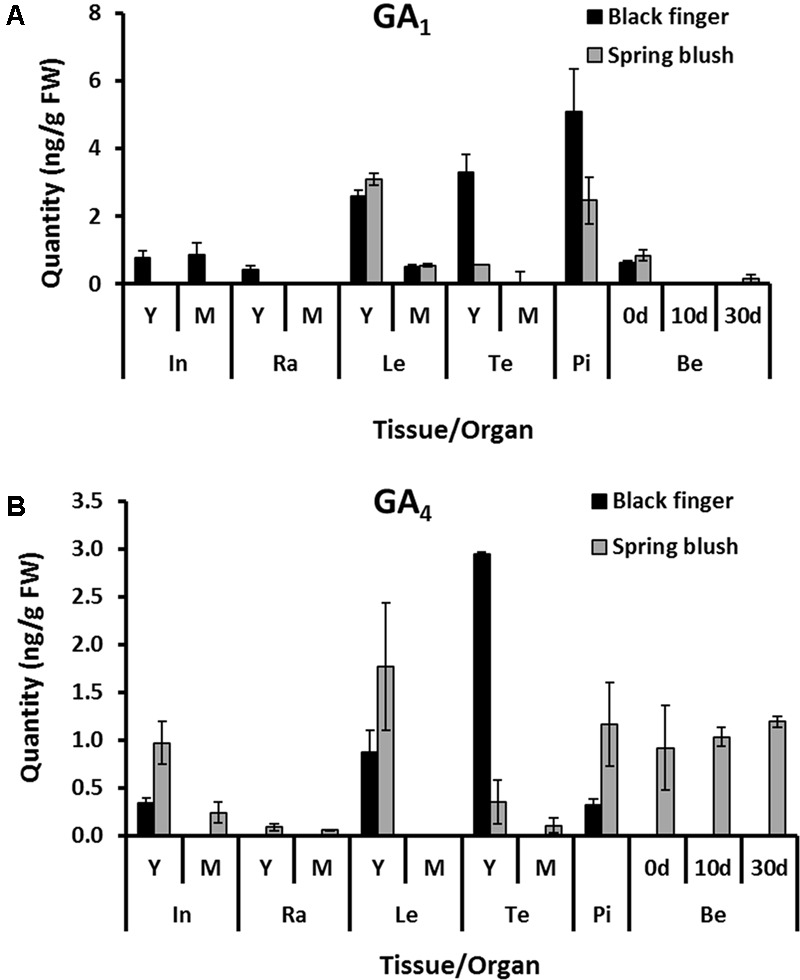
**Spatio-temporal accumulation of endogenous bioactive GAs in *V. vinifera* cv. Black finger (BF) and cv. Spring blush (SB).** Quantification of endogenous GA_1_ **(A)** and GA_4_ **(B)** in organs of BF (black-filled bars) and SB (gray-filled bars) at different developmental stages. Bioactive GAs were extracted from 0.5 g fresh weigh of homogenized tissue, and quantified using triple quadrupole mass spectrometer coupled to an Ultra High Performance Liquid Chromatography (UHPLC) system equipped with an octylphenyl column. The bars represent the mean ± SD. of three biological replicates of extractions. In, internodes; Ra, rachis; Le, leaves; Te, tendrils; Pi, pistils; Be, berries; 0 d, berries sampled at 2–3 mm diameter (E-L 27); 10 d, Berries sampled 10 days after E-L 27; 30 d, berries sampled 30 days after E-L 27; Y, young; M, mature. Samples were collected during the 2010 growing season.

The quantities of endogenous bioactive GA_1_ and GA_4_ are presented in **Figure 7**. The levels of other GA species are presented in Supplementary Table S3 (for internodes, rachis leaves, and tendrils) and Supplementary Table S4 (for pistils and berries). In general, the analysis suggested that: (1) the levels of the different bioactive GA species either decreased or remained constant as organs of both BF and SB developed; (2) except in leaves and berries, GA_1_ levels were higher in young organs of BF than corresponding organs of SB; (3) GA_4_ levels were considerably higher in most organs of SB.

#### Internodes

While GA_4_ was detected in internodes of both cultivars, GA_1_ was present only in BF internodes. GA_4_ was threefold lower in young internodes of BF and undetected in its mature internodes. In young internodes, higher level of GA_8_ (the deactivation product of GA_1)_ was detected in SB, but no significant difference was recorded for GA_34_ (the deactivation product of GA_4_), despite the higher GA_4_ level in SB. GA_8_ was not detected in mature internodes of both cultivars, while GA_34_ was detected in only mature internodes of SB.

#### Rachis

Accumulation of GA_1_ in young rachises of BF was evident, accompanied by significant quantities of GA_8_. In SB, however, both GA_1_ and GA_8_ were not detected in young rachis. In mature rachis, both GA_1_ and GA_8_ were not detected in both cultivars. While both developmental stages of rachis of SB presented GA_4_, it was not detected in young or mature rachis of BF.

#### Pistils and Berries

In pistils of both cultivars, GA_1_ was the more abundant bioactive GA and was twofold higher in BF. GA_4_, on the other hand, was fourfold higher in SB. There was, however, no significant difference between the quantities of both GA_8_ and GA_34_ in pistils of both cultivars. During the pistil-berry transition (fruit set), there was a significant decrease in quantities of GA_1_, which was accompanied by more than twofold increase in GA_8_ accumulation, in both cultivars. As berries of both cultivars developed, GA_1_ quantities dropped to levels below detection, and this was accompanied by a corresponding decrease in GA_8_. Notably, unlike berries of BF, which had no detectable quantity of GA_4_, a steady level of GA_4_ was recorded in the pistils and throughout berry development of SB. A convex profile of GA_34_ was recorded, which peaked at 10 DAF and dropped toward 30 DAF.

#### Leaves and Tendrils

The bioactive GA profiling in other vegetative organs presented notable findings. In young leaves, GA_1_ and GA_8_ levels were comparable in both cultivars but GA_4_ and GA_34_ levels were twofold higher in SB. In young tendrils, however, both GA_1_ and GA_4_ levels were sevenfold higher in BF. In mature tendrils, GA_1_ and GA_4_ were not detected in both cultivars. Deactivation products were higher in BF tendrils.

While GA signaling components are likely to play the central role in mediating the response to GA application, quantities of endogenous bioactive GAs may also modulate the response to GA. It is expected that cultivar with lower level of endogenous bioactive GAs will display stronger response to GA application. However, the varietal differences in total endogenous bioactive GAs were not congruent with the proposed hypothesis and were unable to fully explain the observed varietal differences in the responses of organs to GA.

### SB, the Cultivar with Lower Response to Exogenous GA_3_, Presented Higher Expression of GA Metabolism Genes in Developing Berries

**FIGURE 8:**
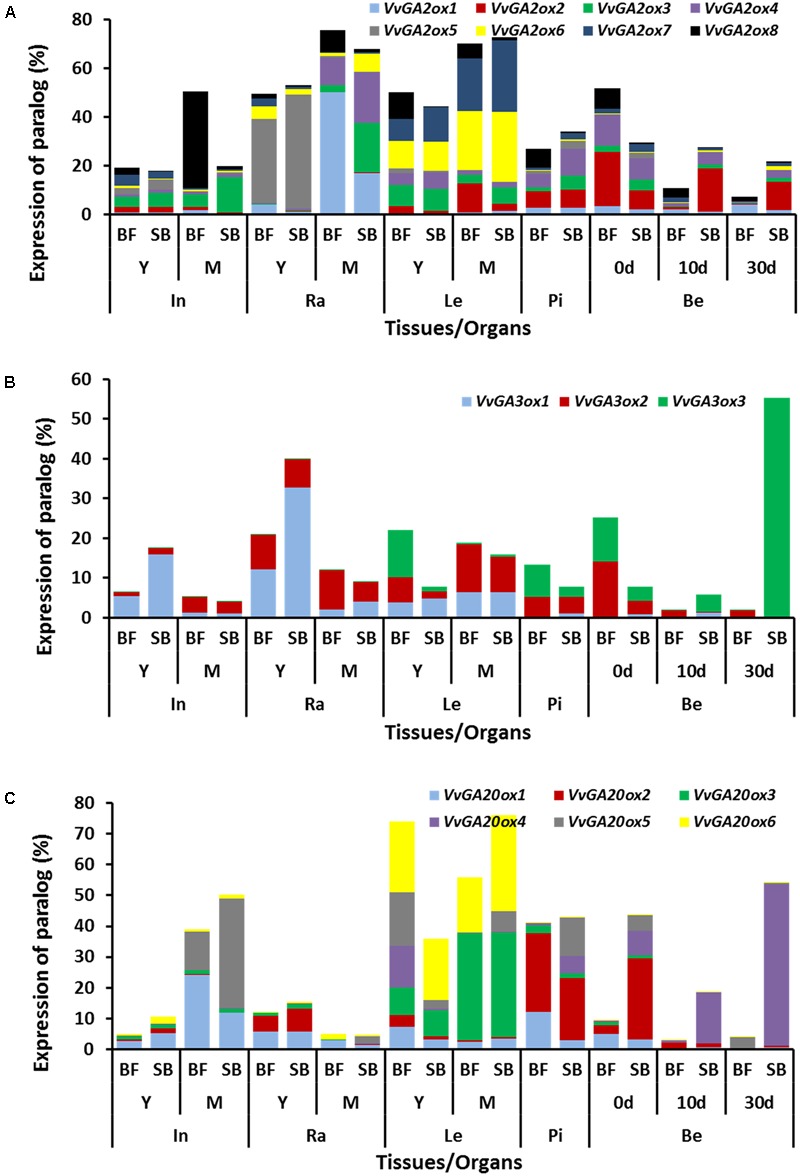
**Spatio-temporal expression profiles of GA metabolism genes in *V. vinifera* cv. Black finger (BF) and cv. Spring blush (SB).** Spatial and temporal expression profiles of *VvGA2ox* **(A)**, *VvGA3ox* **(B)**, and *VvGA20ox* **(C)** paralogs in organs of BF and SB collected during the 2010 growing season. *Y*-axis is the normalized relative expression (NRE) (Giacomelli et al., 2013) of the respective paralog, expressed as a *per cent* of the total NRE of that paralog in all organs analyzed. NREs were calculated, as described by Giacomelli et al. (2013), from the average of three biological replicates of relative transcripts normalized against the expression of *VvGAPDH*, which is unaffected by GA. Transcripts were measured by qRT-PCR using EvaGreen DNA-binding dye on the 96.96 Dynamic Array Integrated Fluidic Circuits (IFCs). In, internodes; Ra, rachis; Le, leaves; Te, tendrils; Pi, pistils; Be, berries; 0 d, berries sampled at 2–3 mm diameter (E-L 27); 10 d, berries sampled 10 days after E-L 27; 30 d, berries sampled 30 days after E-L 27; Y, young; M, mature. Full description of experimental procedure is given in Section “Materials and Methods.” Graphs of the spatial and temporal expression profiles of the individual metabolism genes are presented in Supplementary Figure S5, and detailed statistical significance provided in **Supplementary File S2**.

The significant differences in endogenous GAs in the berries of BF and SB point to potential differences in GA biosynthesis or degradation between the cultivars. To elucidate the molecular sources of the varietal differences in bioactive GA quantities described above, the spatio-temporal transcript levels of the rate-limiting 2-oxoglutarate-dependent dioxygenases (2-ODDs) gene families of grapevine (*VvGA20ox, VvGA3ox, VvGA2ox*) (Giacomelli et al., 2013; Jung et al., 2014) were quantified by qRT-PCR (**Figures 8A–C** and Supplementary Figure S5). For consistency, the nomenclature of genes used in this study is same as reported in our previous study (Giacomelli et al., 2013).

#### Pistils and Berries

In pistils, *VvGA20ox1* expression was 4-fold higher in BF compared to SB, while *VvGA2ox3* and *VvGA2ox4* were 3-fold higher in SB. Substantial quantities of *VvGA20ox5* mRNA were also detected in pistils of SB, but not detected in BF. There were considerable varietal differences in the expressions of *VvGA20ox2, VvGA20ox4, VvGA3ox3, VvGA2ox2, VvGA2ox4*, and *VvGA2ox8* in berries. Compared to BF, SB berries showed higher transcripts of *VvGA20ox2* (8-fold, 0 DAF), *VvGA20ox4* (10- to 60-fold, 0–30 DAF), and *VvGA3ox3* (which was not detected in BF at 10–30 DAF and presented the highest expressed in SB berries, compared to all other tissues). While only one biosynthetic gene was significantly higher in BF berries (*VvGA3ox2:* 4-fold in 0–30 DAF), two deactivation genes had higher transcript levels in this cultivar, compared to SB (*VvGA2ox1*: 2-fold in 0–30 DAF; and *VvGA2ox8*: 6-, 3-, 2-fold in 0, 10, and 30 DAF, respectively). Two additional deactivation genes, *VvGA2ox2* and *VvGA2ox4*, were higher in BF at 0 DAF (3- and 1.3-fold, respectively), and then higher in SB at 10 and 30 DAF (30-fold and 3-fold in SB at 10–30 DAF, compared with BF).

#### Internodes

In young internodes of SB, expression of *VvGA20ox1* and *VvGA3ox1* were 2- to 3-fold higher than in young internodes of BF. In mature internodes of SB, transcript levels of *VvGA20ox5* and *VvGA2ox3* were 3- and 2-fold higher, while that of *VvGA20ox1* and *VvGA2ox8* were 2- and 20-fold lower than in mature internodes of BF.

#### Rachis

In young rachis of SB, *VvGA2ox6* and *VvGA3ox1* mRNA levels were 2- and 3-fold higher than in young rachis of BF. In mature rachis of SB *VvGA2ox3, VvGA2ox4*, and *VvGA2ox6* expressions were 10-, 2-, and 3-fold higher than in mature rachis of BF, while transcript of *VvGA3ox2, VvGA2ox1*, and *VvGA2ox8* were 2-, 3-, and 5-fold higher in mature BF rachis.

#### Leaves

Expressions of *VvGA20ox1, VvGA2ox2* were twofold higher in young leaves of BF, while *VvGA20ox5* and *VvGA3ox2* were, respectively, fivefold and threefold higher in BF.

Some of the GA metabolism genes also displayed potential organ specificity (**Figure 8** and Supplementary Figure S5). *VvGA20ox1* and *VvGA2ox8* were highest expressed in internodes, *VvGA2ox1* and *VvGA2ox5* mainly expressed in rachis, *VvGA20ox3, VvGA20ox6, VvGA2ox6*, and *VvGA2ox7* were mainly expressed in leaves, and *VvGA20ox2, VvGA20ox4, VvGA3ox3, VvGA2ox2* were mainly expressed in pistils and berries.

### Seed Traces Are Present in SB, the Cultivar with Lower Response to GA_3_

**FIGURE 9:**
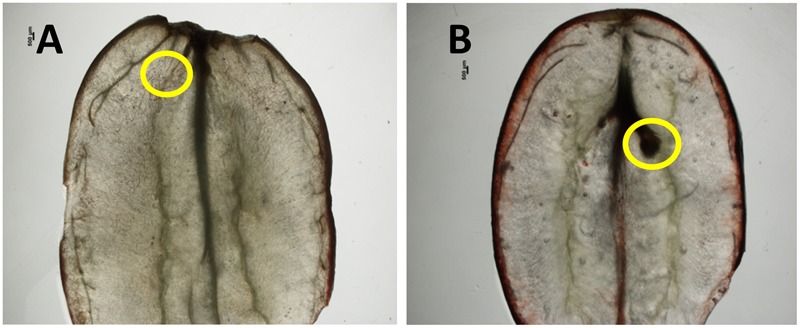
**Seed traces in berries of *V. vinifera* cv. Black finger (BF) and cv. Spring blush (SB).** Anatomy of representative berries of BF **(A)** and SB **(B)**, harvested at 30 days after fruit set, and showing presence or absence of seed trace. Yellow circles indicate position of seed trace. Bar = 500 μm.

Since seed traces of stenospermocarpic cultivar are considered the primary source of GA in the grape berry after endosperm abortion (Conde et al., 2007), it was envisioned that berry variations in bioactive GA content may be influenced by differences in size or presence of seed traces. Analyses of 100 30-day-old berries, sampled randomly from 20 clusters, revealed the presence of seed traces in all berries of SB, while berries of BF had no visible seed traces. Representative berries with these phenotypes are shown in **Figure 9**.

### Comparative Response of Organs of BF and SB to Application of GA_1_ and GA_4_

Based on the fact that different bioactive GA species appear to vary widely in their effects on different plant species, as well as mutants of the same species (Brian et al., 1962; Lange et al., 2005, 2012; Griffiths et al., 2006; Chandler et al., 2008), GA_1_ was identified as the more effective bioactive GA in enlarging berries of certain seedless cultivars (Weaver, 1961; Paleg et al., 1964), and quantities of GA_4_ was higher in most organs of SB than in BF (**Figure 4**), it was speculated that in a specific organ/tissue, the different grapevine cultivars may contain different bioactive GA species, which may also lead to varietal differences in GA response. To this end, young internodes, rachises and berries of both cultivars were treated with GA_1_ and GA_4_. The results show that in both cultivars, application of GA_1_ and GA_4_ produced similar effect in all three organs (Supplementary Figure S6). This is irrespective of the fact that the endogenous levels of these GA species are markedly different in the different organs of both cultivars. Compared to controls, GA_1_ and GA_4_ did not significantly increase internode lengths of BF and SB (Supplementary Figures S6A,B). Both GA_1_ and GA_4_ produced a 3-fold increase in rachis length of BF, and slightly increased the length of SB rachis (Supplementary Figures S6C,D). While the weight of SB berries was unaffected by GA_1_ and GA_4_ application, BF berries were increased by 1.5-fold (Supplementary Figures S6E,F).

## Discussion

In addition to organ/tissue-specific response to GA within a grapevine cultivar (Agüero et al., 2000; Acheampong et al., 2015), varietal-specific differences in organ response have also been reported (Weaver, 1958, Weaver et al., 1962; Agüero et al., 2000; Cheng et al., 2013). Varietal differences in GA response may be a complex phenomenon. Naturally, the primary potential targets for regulation of response are GA metabolism and signaling. However, factors such as penetrability, cell wall composition, cell surface GA receptors, cell number, and cell enlargement capability cannot be discounted. The molecular mechanism regulating these response differences have neither been verified nor explored. As a first step toward understanding this phenomenon, the current comparative study focuses on the potential involvement of GA signaling and metabolism on such differential responses.

### Response of BF to GA_3_ Is Generally Greater than SB

The differential response of berries of BF and SB to GA was based on well-established information from extension service officers and growers, from different growing regions and many growing seasons. To avoid potential local effects, we selected the closest BF and SB vineyards, which shared very similar geographic, topographic and environmental parameters. In addition, we used GA concentrations verified to produce differential response, repeated the experiments over two growing seasons, and calculated responses relative to the triton-treated organs growing in the same vineyard.

The fact that the response of BF to GA_3_ is higher than that of SB in all organs analyzed (**Figure 1**) suggests that varietal-related responses to GA may be regulated by similar mechanisms in both vegetative and reproductive organs. Response to PAC was, however, organ- and cultivar-dependent; with comparable responses recorded for rachis, while response of internodes and berries were higher for BF and SB, respectively. Similar to TS and seeded varieties (Agüero et al., 2000; Acheampong et al., 2015), different organs exhibited different degrees of response to GA and PAC in each cultivar.

The combined PAC-GA treatment was included in the current analysis as a qualitative support to the assumption that the effect observed in the PAC treatment mainly resulted from its effect on GA biosynthesis, since other effects of PAC are well documented (Buta and Spaulding, 1991; Ahmad et al., 2015). However, it is worth noting that the combined PAC-GA treatment present the following complications: (1) two separate treatments are involved in that combined treatment, with a time-lapse between the treatments; (2) the assumption that the PAC pre-treatment completely inhibited GA biosynthesis, and that there was complete exhaustion of endogenous bioactive GAs prior to GA application was not experimentally determined in these grapevine cultivars. Without prior knowledge regarding the rate and efficiency of the inhibition and degradation of endogenous GAs, the response of organs after PAC-GA treatments may reflect a combination of unknown quantities of endogenous GA and known concentration of the applied GA. Therefore, we did not use data from this treatment regime to analyze quantitative variations in response of both cultivars; thus, avoiding over-simplified quantitative consideration which may be biologically misleading.

It is important to note that while berries of SB appear to be non-responsive to GA, compared to BF, SB cannot be considered a GA insensitive variety, since the rachis and internodes respond to GA (**Figure 1**).

### Varietal Differences in GA_3_ Response Could Not Be Attributed to Specific Bioactive GA Species

Similar to Arabidopsis (Griffiths et al., 2006) and pumpkins (Lange et al., 2005, 2012), we found high levels of GA_4_ in most organs of both varieties; suggesting that it is the major bioactive GA regulating growth in grapevines. Even though specific GA species have been reported to elicit growth of specific organs in grapevine and other plant species (Paleg et al., 1964; Kato et al., 1998; Ross et al., 2000; Spielmeyer et al., 2002; Wolbang et al., 2004; Griffiths et al., 2006; Hu et al., 2008), our results of on-field experiment show that both GA_1_ and GA_4_ affect organ growth similarly (Supplementary Figure S6), suggesting that the differential response does not involve differences in perception of a specific bioactive GA.

### Varietal Differences in GA_3_ Response Was Not the Result of Allelic Variation of Signaling Components

When signaling components are considered as the potential source for differential response, both their availability (quantitative differences) and proper biological function (qualitative changes) have the potential to regulate the response. Quantitative and qualitative determinants to GA response have been reported in GA response mutants of model plants (Dill et al., 2001; King et al., 2001; Sasaki et al., 2003; Griffiths et al., 2006; Hirano et al., 2010; Yamamoto et al., 2010). In the current comparative study, Y2H assays showed that different cultivar-specific alleles of VvDELLA proteins (Supplementary Figure S2) did not differ in their interaction with VvGID1s or VvSLY1s (**Figure 2**). Moreover, immunoblot analyses verified GA-dependent VvDELLA1 and VvDELLA2 protein degradation in various organs (**Figure 5**), suggesting that allelic differences between varieties neither affects the nature of biological activity nor results in detectible perturbation of the degradation machinery of VvDELLA proteins in response to GA *in planta*.

### Varietal Differences in Response to GA_3_ May Be a Consequence of Differences in Quantity of GA Signaling Components

Marked differences in quantities of VvDELLA were recorded in young organs of BF, compared to SB (**Figure 4**). Differences in quantities of DELLA were suggested as the cause for differences in response to GA between Arabidopsis ecotypes. *Col-0* displayed a more severe fertility phenotype than L*er*. Additionally, *Col-0 rga gai* mutant was entirely male sterile while the equivalent L*er* mutant was fertile. It was suggested that differences in quantities of RGL1, RGL2, and RGL3 was responsible for the differential response (Plackett et al., 2014). It was also shown that rescue of microspore development in GA-deficient *ga1-3*, required knockout of three DELLAs (RGA, RGL1, RGL2) in L*er* (Cheng et al., 2004), while knockout of RGA alone was sufficient in *Col-0* (Tyler et al., 2004). This may suggest higher level of RGL1 and RGL2 in L*er*. Marked reduction of DELLA proteins level resulted in enhanced growth also in a GA deficient *ga1-3* background, supporting its central role in growth response (Dill and Sun, 2001). Here, it is suggested that the differences in quantities of VvDELLA in BF and SB may be the main factor regulating the varietal differences in organs response to GA application. It is assumed that the higher VvDELLA levels in young organs of BF results in greater repression of GA-mediated growth, and its degradation, through GA application, results in greater growth response in organs of this cultivar, compared to SB, as further detailed in the Section “Integrative Working Hypothesis of Potential Factors Influencing BF Behavior.”

The significant accumulation of all three VvDELLA in young BF organs could be due to: (1) higher transcription or translation or post-translational modification of all three proteins; (2) lower endogenous bioactive GAs; (3) decreased efficiency of the GA-induced proteolytic degradation mechanism of VvDELLA proteins. Expression data (**Figure 3**) does not support the first assumption as *VvDELLA* transcripts in most organs appear higher in SB. It is unlikely that factors that affect translation *in cis* will be similarly mutated in all three genes. However, the probability of a mutation in a regulator that affect translation or post-translational modification *in trans* cannot be discounted. Higher or comparable levels of bioactive GAs in most BF organs, apart from leaves and berries, rules out the second scenario as a probable primary cause. The fact that application of GA induced VvDELLA1 and VvDELLA2 degradation suggests that the VvDELLA degradation machinery is functional in BF. Thus, we assume that the most likely cause of the high accumulation of all three VvDELLAs in young BF organs may be lower efficiency of the VvDELLAs degradation, a mechanism that is expected to be shared by all three VvDELLAs. Such limited efficiency may be the result of lower quantities of a modulator required specifically for VvDELLA degradation.

Interestingly, in mature organs from both cultivars there was no consistent difference in VvDELLA protein levels. As previously suggested for TS (Acheampong et al., 2015), it is possible that as organs mature and growth rate declines, VvDELLAs do not play significant roles in regulation of organ growth, and their quantities in this developmental stage may not reflect the varietal differences in response of young organs.

### The Potential Role of VvSLY1 as a Trigger for the Varietal Differences in VvDELLA Levels

The natural suspect is VvSLY1, whose role in GA-mediated DELLA degradation and organ response has been demonstrated in model plants (McGinnis et al., 2003; Sasaki et al., 2003). Compared to wild type, *sly1-10* (Arabidopsis) or *gid2* (rice) loss-of-function mutants were shown to accumulate more DELLA proteins (McGinnis et al., 2003; Sasaki et al., 2003; Dill et al., 2004). In agreement, a significantly lower level of *VvSLY1b* transcript was recorded in BF, compared to SB and TS. The grapevine genome uniquely encodes two functional VvSLY1 homologs while the genomes of other angiosperms have been reported to carry single SLY1 gene. However, we assume that *VvSLY1b* rather than *VvSLY1a* has a role in regulating the varietal differences in VvDELLA accumulation and hence GA response in the analyzed organs. This assumption is based on that fact that: (1) while *VvSLY1b* transcript was significantly higher in SB organs, *VvSLY1a* was only marginally higher in young rachis and berries of SB, and presented no significant difference in young internodes (**Figures 6A,B**); (2) current Y2H data (**Figure 2A**) and results from our previous publication (Acheampong et al., 2015) indicate that interaction between VvSLY1a and VvDELLA proteins is at least sevenfold less than interactions between VvSLY1b and VvDELLA proteins. As VvSLY1b has a stronger affinity for all three VvDELLA proteins than VvSLY1a, varietal differences in its expression may significantly affect the degradation efficiency of all VvDELLA. It should, however, be stated that, in the absence of solid experimental data, the contribution of VvSLY1a to varietal differences in GA response cannot be completely discounted.

In light of the above, the suggested hypothetical scenario is that relatively low availability of VvSLY1b in young organs of BF results in fewer VvDELLA–VvSLY1b complexes, thus decreasing efficiency of polyubiquitination and degradation by the 26S proteasome, and increasing VvDELLA accumulation in these organs. In support of this hypothesis, Arabidopsis *sly1-d* mutants, with enhanced DELLA–SLY1 interaction than wild type, accumulated less DELLA proteins and enhanced GA signaling in *rga-Δ17* mutant lines (Dill et al., 2004). The observed varietal difference in *VvSLY1b* transcript could be due to mutation(s) in an element which regulate transcription from the *VvSLY1b* promoter, *in cis* or *in trans.* The nature of the difference is yet unclear and will require further analyses.

### The Consequences of Higher Expression of *VvGID1*

In addition to lower *VvSLY1b* transcript and higher VvDELLAs, BF presented higher transcript levels of the GA receptors, *VvGID1*s, suggesting availability of more receptor molecules and thus greater response of organs to GA. In support, rice lines over-expressing *GID1* showed higher response to GA_3_ application than wild type controls (Ueguchi-Tanaka et al., 2005). Additionally, variations in phenotype of Arabidopsis mutants were attributed to the differential expression of Arabidopsis *GID1* homologs (Suzuki et al., 2009).

The observed varietal differences in the *VvGID1* transcripts in BF and SB could have resulted from differences in levels of bioactive GA, differences in VvDELLA accumulation, or both. Support for endogenous bioactive GA regulation of *GID1* expression by negative feedback was formerly presented (Ueguchi-Tanaka et al., 2005; Griffiths et al., 2006; Li et al., 2011; Voegele et al., 2011; Acheampong et al., 2015). While we observed inverse correlation between GA_4_ levels and *VvGID1b* transcripts in organs of SB and BF, levels of GA_1_ did not show the same trend. In addition, no differential growth response was observed in different organs upon application of GA_1_ and GA_4_ to BF and SB (Supplementary Figure S6). Hence, our data is only consistent with DELLA-mediated regulation of *VvGID1* expression. Solid support for the latter can be drawn from findings in Arabidopsis (Cao et al., 2006; Griffiths et al., 2006) and rice (Ueguchi-Tanaka et al., 2008) in which GID1 transcripts were upregulated by DELLA. Since GA signaling is highly conserved in higher plants (Harberd et al., 2009; Sun, 2010), it is likely that a similar scenario may be occurring in grapevine.

### Absence of Seed Traces in BF May Result from GA_1_-Induced Fruit Set and Limit GA Level in the Berry

The variations in size of seed traces in stenospermocarpic varieties is primarily determined by the genotype and also affected by environmental factors (Cabezas et al., 2006; Doligez et al., 2013). The absence of visible seed traces in BF suggests that it is in the smallest end of that size spectrum. Alternatively, it may suggest that fruit set in BF is induced without fertilization, despite its stenospermocarpic genetic background. This could be due to the uniquely high level of GA_1_ in the pistils (**Figure 7**). Indeed, in stenospermocarpic varieties, GA induces fruit set in a fertilization-independent manner when flowers are emasculated at least 2 weeks before anthesis and treated with GA_3_ (Or et al., unpublished). Support for GA_1_ induced parthenocarpy can be drawn from studies showing that GA_1_ level was higher in tomato *pat-3/pat-4* parthenocarpic mutants, compared to wild types (Fos et al., 2001). Moreover, application of GA_1_ led to induction of parthenocarpic berries in seeded grapevine cultivars (Kato et al., 1998), and resulted in parthenocarpic growth of unpollinated Madrigal tomato carpels (Fos et al., 2000). The reason for the high GA_1_ in BF is yet unclear. However, in view of the uniquely high VvDELLA accumulation in this cultivar, causal link should be considered. Modifications in bioactive GA quantities resulting from high DELLA accumulation have been reported in mutants of grapevines and other species (Boss and Thomas, 2002; Chandler et al., 2002; Itoh et al., 2005; Busov et al., 2006; Griffiths et al., 2006; Boccaccini et al., 2014).

Interestingly, varietal differences in response to PAC and total endogenous bioactive GAs measured were not congruent with varietal differences in the responses of different organs of BF and SB to GA. In berries, however, both growth response to PAC and endogenous bioactive GA measurements correlated with the response to GA. As suggested above, the lower level of GA in BF, which may further contribute to its high response to GA application, can be associated to the absence of seed traces, which are the main sources of bioactive GAs in stenospermocarpic cultivars (Conde et al., 2007). In agreement with this hypothesis, (1) the absence of seed traces in BF correlated with low levels of bioactive GA (both GA_1_ and GA_4_) in the berries; (2) the presence of seed traces in the berries of SB was accompanied by higher bioactive GA_4_ quantities, possibly resulting from the upregulation of GA biosynthetic genes, *VvGA20ox4* and *VvGA3ox3* (**Figure 9**). It is important to note that even though berries of SB do not respond to GA, this cannot be considered a GA insensitive variety since the rachis and internodes clearly respond to GA (**Figure 1**). Alternatively, it can be considered as normal/less responsive variety, compared to BF. Yet, sufficient endogenous GA produced by its seed rudiments during berry development may be the cause for the lack of response of its berries to GA.

### Integrative Working Hypothesis of Potential Factors Influencing BF Behavior

While both BF and SB have a functional DELLA degradation machinery in the presence of GA, there are significant differences in quantities of central components of the GA signaling cascade in these cultivars. Our results show that, compared to SB, BF (1) presents higher response to GA; (2) accumulate very high amounts of all three VvDELLAs; (3) presents high and low transcripts of *VvGID1* and *VvSLY1b*, respectively. Such coordinated and significant differences in quantities of central components of GA-VvGID1-VvDELLA-VvSLY complex, which directly regulates VvDELLA degradation, raise the hypothesis that the difference in the number of the complexes formed may be the primary factor regulating the observed GA-response phenotypes in organs of both cultivars. The following scenario is proposed to account for the differences in response: low quantities of bioactive GA in untreated organs results in limited number of GA-VvGID1-VvDELLA-VvSLY complexes, despite the availability of VvGID1 and VvSLY1b. Therefore, VvDELLA degradation is limited and DELLA proteins accumulate and are active. When GA is applied, and VvGID1 and VvSLY1b are available, VvDELLA degradation is enhanced due to increased formation of GA-VvGID1-VvDELLA-VvSLY. The higher response of BF organs, compared to SB, is the result of modified behavior in both situations. Under limited GA availability, the number of GA-VvGID1-VvDELLA-VvSLY complexes is further limited, due significantly lower level of *VvSLY1b.* When GA is applied, VvDELLA degradation and inactivation are further enhanced due to (1) higher probability of GA-VvGID1-VvDELLA-VvSLY formation in the presence of higher level of VvGID1; (2) increased formation of GA-VvGID1-VvDELLA, which suppress DELLA action by non-proteolytically blocking of the transcriptional activity of DELLA (Ariizumi et al., 2008; Hauvermale et al., 2014). These modifications in availability of GA-VvGID1-VvDELLA-VvSLY complexes in BF result in enhanced growth inhibition, when GA is limited, and enhanced growth response when GA is supplied.

Alternatively, it can be argued that higher number of cells in untreated organs of BF is responsible for its higher response to GA. Difference in cell enlargement capability may also be considered: where cell expansion is limited in SB or enhanced in BF. However, such assumptions do not account for the smaller size of untreated BF organs and their slower growth rate as compared to SB. In addition, the varietal differences in quantities of central GA signaling components are not unidirectional.

The remarkable differences detected in GA response and levels of central GA signaling components among varieties of this perennial crop, which is not often analyzed thoroughly, expose both the complexity of the system and its strong reliance on the mechanisms discovered in model plants.

## Author Contributions

AA, AL, and EO contributed to the conception and design of the work. AA, CZ, TH, LG, YT, YJ, and YK involved in acquisition and analysis of data. AA, LG, YJ, AL, and EO were involved in drafting the manuscript. All authors were involved in final approval of version submitted. All authors are accountable for all aspects of the work relating to accuracy and integrity.

## Conflict of Interest Statement

The authors declare that the research was conducted in the absence of any commercial or financial relationships that could be construed as a potential conflict of interest.

## Footnotes

1 http://www.clontech.com/US/Support/xxclt_onlineToolsLoad.jsp?citemId=https://www.takara-bio.co.jp/infusion_primer/infusion_primer_form.php&section=16260&xxheight=1800

2 http://www.genoscope.cns.fr/spip/Vitis-vinifera-e.html

## Supplementary Material

FILE S2 **Statistical analyses for expression profiles of GA signaling and metabolism genes**.

## Abbreviations

BF: Black finger
DAF: days after fruit set
GA: gibberellins
PAC: paclobutrazol
SB: Spring blush
VvGID1: *Vitis vinifera* GIBBERELLIN-INSENSITIVE DWARF1
VvSLY1: *Vitis vinifera* SLEEPY1
